# S100A8 promotes migration and infiltration of inflammatory cells in acute anterior uveitis

**DOI:** 10.1038/srep36140

**Published:** 2016-10-27

**Authors:** Yuqin Wang, Zuhui Zhang, Laihe Zhang, Xinxin Li, Rui Lu, Peipei Xu, Xuhong Zhang, Mali Dai, Xiaodan Dai, Jia Qu, Fan Lu, Zailong Chi

**Affiliations:** 1 Laboratory of Neurovascular Biology, School of Ophthalmology and Optometry and the Eye Hospital of Wenzhou Medical University, Wenzhou, China; 2 The State Key Laboratory Cultivation Base and Key Laboratory of Vision Science, Ministry of Health, Wenzhou, China

## Abstract

Uveitis, the pathologic condition of inflammation of the uvea, frequently leads to severe vision loss and blindness. S100A8 is a calcium-binding protein which mainly expresses in granulocytes and monocytes and plays a prominent role in the regulation of inflammatory processes and immune response. Here, we determined the role of S100A8-positive cells in acute anterior uveitis (AAU) and keratitis. In rat models of endotoxin (lipopolisaccharide, LPS) -induced uveitis (EIU) and keratitis, S100A8-positive granulocytes and monocytes increased significantly in the iris-ciliary body and cornea as well as in the blood. Interestingly, Glucocorticoids slightly increased S100A8 levels in leukocytes, but reduced its presence significantly in the iris-ciliary body after LPS injection. Moreover, inhibition of NF-kB activation remarkably suppressed both progression of AAU and total S100A8 levels in leukocytes and the iris-ciliary body after LPS administration. Additionally, S100A8 protein level was also found to be elevated in the serum of AAU patients parallel with the progression of AAU through the designated clinical stages. Thus, S100A8 plays a pivotal role in the processes of AAU through involvement in migration and infiltration of S100A8-positive cells. Our findings suggest that serum levels of S100A8 protein can be used to monitor inflammatory activity in AAU.

As a member of the calcium-binding S100 protein family, S100A8 (also known as MRP8, calgranulin A, or CP-10 in mice) is a 10.8 kD low molecular weight protein equipped with an EF hand motif, which binds Ca^2+^ selectively and with high affinity. Only after Ca^2+^ binding can S100A8 perform many of its recognized functions. S100A8 is specific for cells of myeloid origin such as granulocytes, monocytes, and macrophages, but it is not detected in resident tissue macrophages^1^,^2^,^3^,^4^,^5^,^6^. S100A8 is overexpressed in various inflammatory and infectious pathologies including rheumatoid arthritis, Crohn’s disease, psoriasis, cystic fibrosis, *Pseudomonas aeruginosa* keratitis, and autoinflammatory diseases^7^,^8^,^9^,^10^,^11^,^12^,^13^. S100A8 and S100A9 may have different functions in mice and humans; however, Ca^2+^ can induce formation of stable heterodimers of S100A8/S100A9. The dimer is also known as Calprotectin. In human myeloid cells, S100A8/S100A9 is co-expressed as a heterodimer, and this dimer represents 45% of the cytosolic protein in neutrophils^3^. Targeted disruption of S100A8 in mice caused lethality early in development^14^. In contrast, mice lacking S100A9 are viable, fertile, and are overall healthy with a mild peripheral neutropenia^15^,^16^. S100A8/S100A9 proteins are produced intracellularly in granulocytes and monocytes, and then released extracellularly. Extracellular S100A8/S100A9 is thought to influence multiple steps in the leukocyte-recruitment process via binding to surface receptors including Toll-like receptor-4 (TLR4)^12^,^17^ and the receptor of advanced glycation endproducts (RAGE)^13^. This results in the induction of proinflammatory cytokine secretion via activation of transcription factor NF-kB^18^,^19^,^20^. S100A8 has anti-oxidant activity and regulates adhesion of neutrophils and monocytes through control of integrin activity^21^,^22^,^23^,^24^. Also, studies performed by Hsu *et al*. have reported on regulation of S100A8 by glucocorticoids^25^.

Uveitis is inflammation of the uveal tract of the eye which includes the iris, ciliary body and choroid. It is a leading cause of blindness worldwide. Causes of uveitis include infection, injury, autoimmune or inflammatory diseases; however in many cases the exact cause is unknown. Anterior uveitis is the most common form, accounting for between 50% and 90% of uveitis cases in western countries and between 28% and 50% of cases in Asian countries^26^. Acute anterior uveitis (AAU) involves a specific kind of immune inflammation which is associated with numerous systemic diseases such as rheumatoid arthritis. The pathology is acute and often recurrent, features which are significantly associated with high morbidity and blinding. Walscheid *et al*.^27^ found that among juvenile idiopathic arthritis-associated uveitis (JIAU) patients with autoimmune uveitis (a common chronic anterior uveitis), S100A8/A9 and S100A12 levels were increased in the serum and aqueous humor. These serum levels reflect activity of joint and eye disease. Proteomic analysis of rat models demonstrated that S100A8 and S100A9 were elevated in the aqueous humor in both experimental autoimmune uveitis (EAU) and primed mycobacterial uveitis (PMU)^28^. Systemic injection of LPS induces bilateral acute ocular inflammation in susceptible strains of rats and mice, which is an animal model for human AAU^29^. The inflammatory response in endotoxin-induced uveitis (EIU) peaks 24 hours after LPS injection and subsides within the next 48 hours in experimental animals^30^,^31^,^32^. LPS induces not only uveitis but also keratitis, closely representing the physiologic disease condition in humans^33^,^34^,^35^. We have previously shown that S100A9 is linked with inflammation in EIU models and that application of an NF-kB blocker inhibited the LPS-driven upregulation of S100A9^35^.

To date, there have been few studies on S100A8 and uveitis, and the extracellular function of this protein in AAU remains unclear. Here, we report the role of S100A8 in anterior ocular inflammation and the effect of glucocorticoids and NF-kB inhibitors on S100A8 expression. In addition, our study tracks circulating levels of S100A8 protein through the different stages of AAU in patients. Our findings suggest that S100A8 is involved in the pathological inflammation of EIU and keratitis, and that measuring S100A8 levels can be a useful method to monitor inflammatory activity in AAU.

## Results

### Changes in S100A8 expression in circulating blood leukocytes and the iris-ciliary body after LPS treatment

**Figure 1:**
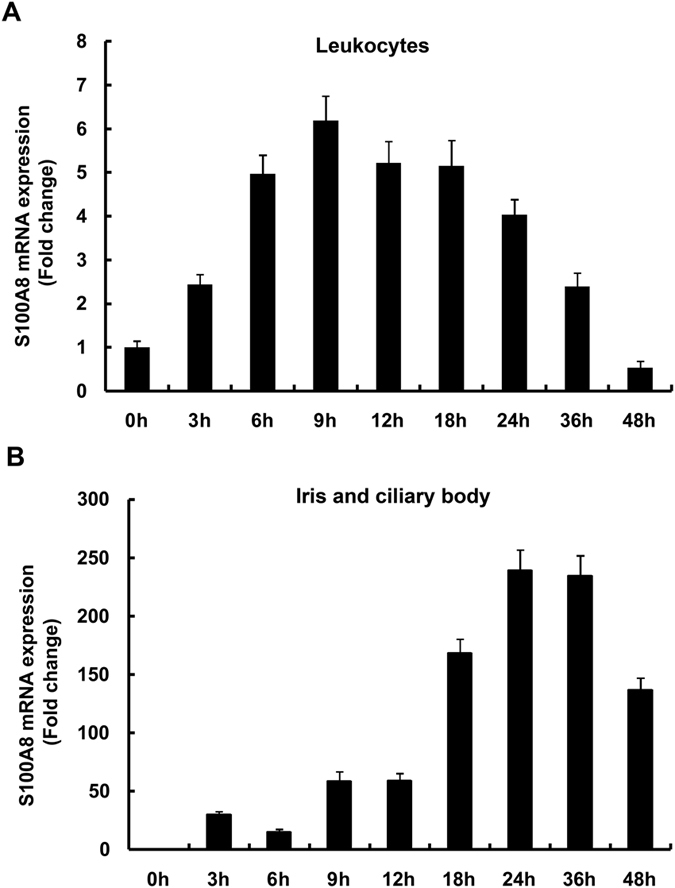
Time course of S100A8 expression changes following LPS injection. (**A**) Leukocyte S100A8 mRNA. (**B**) Iris-ciliary body S100A8 mRNA. Mean ± SD (N = 8) are shown.

In order to understand the changes in S100A8 mRNA expression in EIU rat models, we extracted total RNAs from circulating leukocytes and the iris-ciliary body and performed quantitative real-time PCR. After injection of LPS, mRNAs for S100A8 in circulating blood leukocytes increased at 3 hours post-injection, reached maximum (6.2 ± 0.6 fold) at 9 hours post, and then decreased gradually (Fig. 1A). In contrast, S100A8 mRNAs in the iris-ciliary body increased at 9 hours post-injection, reached maximum (240 ± 17 fold) at 24 to 36 hours post, and then gradually decreased (Fig. 1B). This delay in the expression of S100A8 mRNAs in the iris-ciliary body compared to the blood suggests the possibility that circulating leukocytes are migrating from the blood and infiltrating the iris-ciliary body.

**Figure 2:**
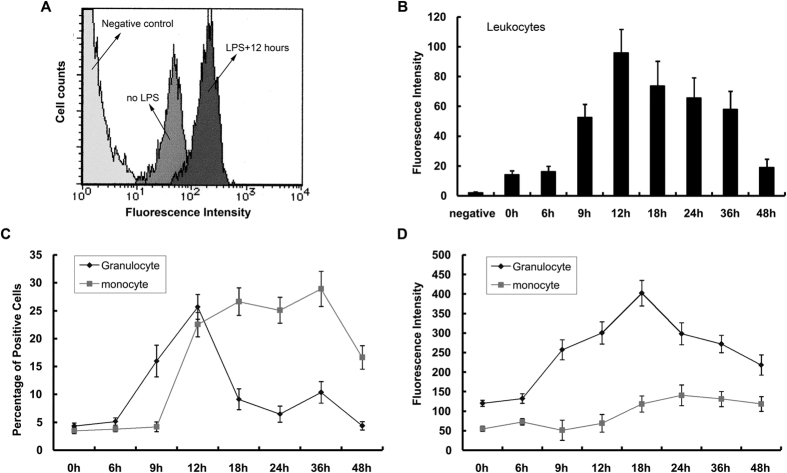
Flow cytometric analysis of intracellular S100A8 expression in circulating leukocytes. (**A**) Fluorescence Intensity histograms of leukocyte S100A8 expression in cells from negative control, untreated, and 12 hours post-LPS injection. (**B**) Time course of S100A8 fluorescence intensity in circulating leukocytes following LPS-injection. (**C**) Percentage of S100A8-positive cells in granulocyte and monocyte populations following LPS injection. (**D**) S100A8 fluorescence intensity in granulocyte and monocyte populations following LPS injection. Granulocytes and monocytes were differentiated by co-staining with anti-granulocyte and anti-CD14 antibodies, respectively. Mean ± SD (N = 4) are shown.

Flow cytometric analysis showed that levels of S100A8 proteins in circulating blood leukocytes increased and reached maximum at 12 hours after LPS injection (Fig. 2A). Before LPS injection, fluorescein intensity was very low (14 arbitrary units) in leukocytes. In contrast, post-LPS injection we observed that the fluorescein intensity in circulating leukocytes increased at 9 hours, reached maximum (96 arbitrary units) at 12 hours, maintained that level for 36 hours, and then gradually decreased (Fig. 2B). After LPS injection, percentages of S100A8-positive granulocytes increased at 9 hours, reached a maximum (25.7 ± 2.2%) at 12 hours, and then decreased (Fig. 2C), whereas in contrast, percentages of S100A8-positive monocytes increased to 23–29% at 12–36 hours after LPS administration (Fig. 2C). These results indicated that granulocytes induced S100A8 expression in response to LPS more quickly than monocytes. On the other hand, expression levels of S100A8 proteins in granulocytes was higher than monocytes at all time points, with or without LPS administration (Fig. 2D).

### S100A8-positive cells infiltrate into the iris-ciliary body and cornea after LPS injection

**Figure 3:**
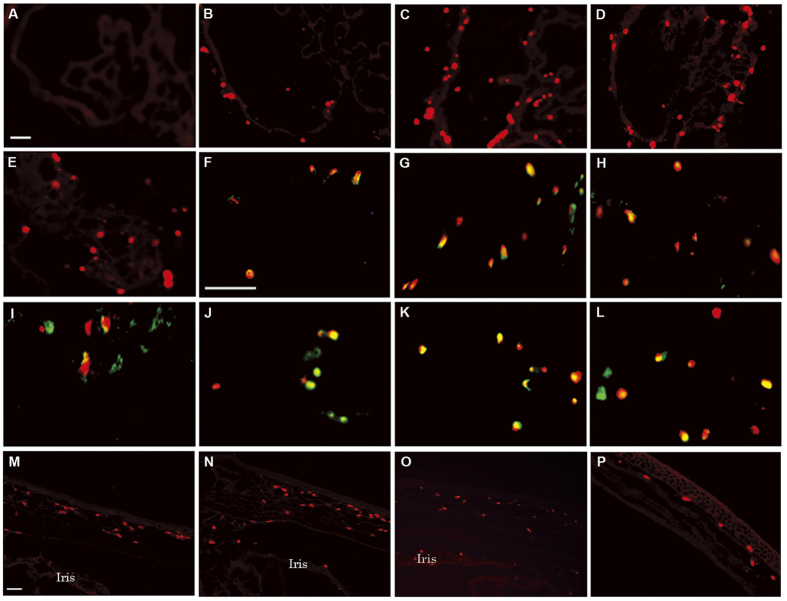
S100A8 immunolocalization in the iris-ciliary body and cornea. (**A–E**) Immunofluorescent staining for S100A8 (red) 24 hours after injection of (**A**) saline; and (**B**) 18, (**C**) 24, (**D**) 36, and (**E**) 48 hours after injection of LPS into the iris-ciliary body. **(F–I)** Double labeling of S100A8 (red) and granulocytes (green) at (**F**) 18, (**G**) 24, (**H**) 36, and (**I**) 48 hours post LPS injection. **(J–L)** Double labeling of S100A8 (red) and monocytes (green) at (**J**) 24, (**K**) 36, and (**L**) 48 hours after LPS injection. (**M–P**) Immunofluorescent staining of S100A8 positive cells (red) in the peripheral cornea at (**M**) 24, (**N**) 36, and (**O**) 48 hours post-LPS injection. (**P**) S100A8 in the central cornea 48 hours after LPS treatment. Bars indicate 50 μm. Results are representative of experiments using 4 separate pairs of eyes in control and LPS-treated groups.

No cells in the iris-ciliary body express S100A8 in the absence of LPS (Fig. 3A). S100A8-positive cells (red) were present at 18 hours (Fig. 3B), increased in number at 24–36 hours (Fig. 3C,D), and decreased at 48 hours (Fig. 3E). Immunofluorescent staining shows the co-localization of S100A8 and granulocyte markers at 18–48 hours in the same tissue (Fig. 3F–I). Yellow indicates S100A8-positive granulocytes. The number of S100A8-positive granulocytes increased at 18 hours (Fig. 3F), reached a maximum at 24–36 hours (Fig. 3G,H), and reduced at 48 hours (Fig. 3I), appearing similar to the expression pattern of S100A8 mRNAs in the iris-ciliary body (Fig. 1B). After LPS injection, a small number of cells in which S100A8 co-localized with CD68 (monocytes/macrophages) were present at 24 hours (Fig. 3J). These cells, which distributed predominantly at the base of the iris-ciliary body, increased in numbers at 36 hours (Fig. 3K) and 48 hours (Fig. 3L), and disappeared at 72 hours (data not shown). The expression pattern of S100A8-positive cells in the anterior part of the uvea is similar to that of circulating leukocytes. Consistently, migration and infiltration of granulocytes responded to LPS faster than did monocytes. S100A8 protein does not express in resident tissue macrophages as evidenced by labeling with ED2 antibody (data not shown).

After LPS injection, S100A8-positive cells (red) were found in the peripheral cornea at 24 and 36 hours (Fig. 3M,N). 48 hours after LPS injection, S100A8-positive cells were reduced in the peripheral cornea (Fig. 3O) but increased in the central cornea (Fig. 3P). S100A8-positive cells disappeared from the cornea at 72 hours (data not shown).

### Glucocorticoids in S100A8 expression

**Figure 4:**
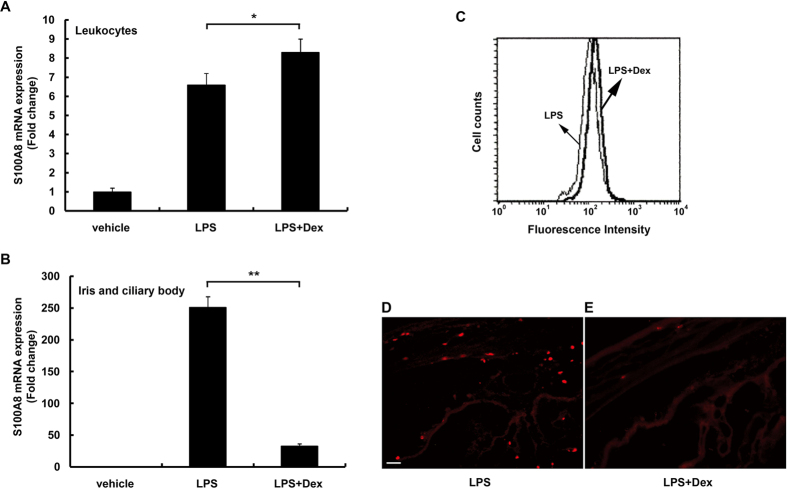
S100A8 levels in circulating leukocytes and the iris-ciliary body after injection of DEX and LPS. (**A**) S100A8 mRNA levels in blood leukocytes 9 hours after injection of LPS or LPS + DEX. (**B**) S100A8 mRNA levels in the iris-ciliary body 24 hours after the injection of LPS. (**C**) S100A8-positive leukocytes 12 hours after the injection of LPS with or without DEX. (**D,E**) Immunofluorescent staining for S100A8 36 hours after the injection of LPS in the iris-ciliary body without (**D**) and with DEX (**E**). Mean ± SD (n = 4 pairs of eyes) are shown. *p < 0.05; **p < 0.01 compared to LPS using *t*-test. Bars indicate 50 μm.

Glucocorticoids are widely used to treat inflammatory and autoimmune diseases including uveitis. Based on our previous research^35^, 1 mg/kg of DEX was injected 30 min before LPS treatment in the rat EIU model. Interestingly, our new data showed the level of S100A8 mRNA to be increased in circulating blood leukocytes (Fig. 4A), but significantly reduced in the iris-ciliary body (Fig. 4B). Flow cytometric analysis also demonstrated that DEX slightly increased the level of S100A8 protein in circulating blood leukocytes in EIU rats (Fig. 4C), paralleling the expression changes seen in mRNA levels. In contrast, at 36 hours post-LPS injection DEX markedly reduced the number of S100A8-positive cells in the iris-ciliary body (Fig. 4E) compared to those groups without DEX treatment (Fig. 4D). Thus, DEX strongly inhibited migration and infiltration of inflammatory cells from the blood into the anterior uvea, but did not inhibit the expression of S100A8 in circulating blood leukocytes.

### Effect of BAY 11-7085 on S100A8 expression in EIU

**Figure 5:**
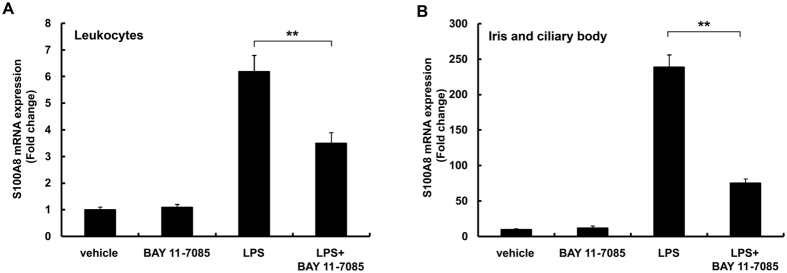
Cell-specific S100A8 expression after injection of BAY 11-7085 and LPS. (**A**) S100A8 mRNA levels in circulating blood leukocytes and (**B**) iris-ciliary body. BAY 11-7085, 10 mg/kg, or vehicle was administered intraperitoneally at the same time as LPS injection. Mean ± SD (n = 4 pairs of eyes) are shown. **p < 0.01 compared to LPS using One Way ANOVA, Tukey’s *post-hoc* test.

Following LPS injection, protein content in the aqueous humor increased at 6 hours, reached a maximum at 18 to 36 hours, and then gradually decreased, as we have previously shown^35^. Intracameral cells also increased at 12 hours and reached maximal levels at 18–36 hours in EIU rats^35^. NF-kB plays a key role in inflammatory and autoimmune diseases. BAY 11-7085 is an inhibitor of I-kB phosphorylation, widely used to block activation of NF-kB. Our previous data demonstrated that BAY 11-7085 significantly inhibited the accumulation of intracameral protein and cells at 24 hours after LPS treatment^35^. Similar to the suppression of inflammation seen in the anterior uvea, BAY 11-7085 greatly suppressed the expression of S100A8 mRNA in circulating leukocytes at 9 hours (Fig. 5A) and in the iris-ciliary body at 24 hours (Fig. 5B) after LPS injection. No accumulation of protein or cells in the anterior chamber was found in rats treated with BAY 11-7085 alone. Our data suggests that a possible mechanism of BAY 11-7085-mediated inhibition of migration and infiltration of inflammatory cells from blood into ocular tissue may be via inhibiting the expression of S100A8 in circulating leukocytes.

### Serum levels of S100A8 protein in AAU patients

**Figure 6:**
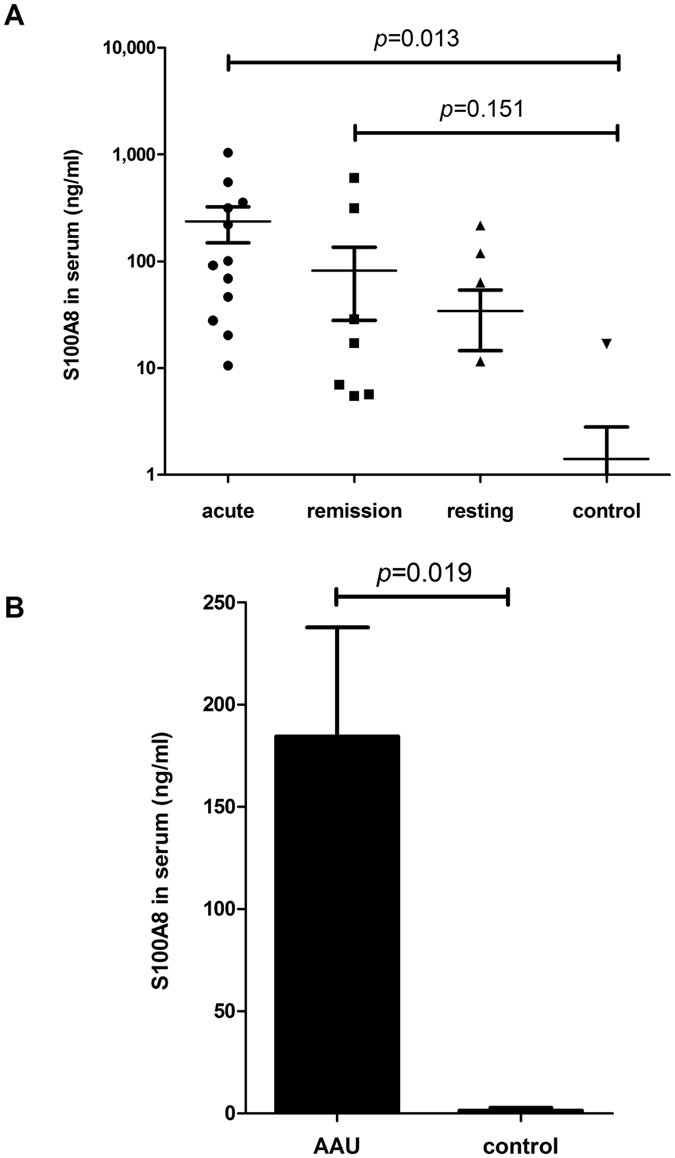
Serum S100A8 levels in AAU patients. (**A**) S100A8 protein levels in control (n = 29) and acute (n = 12), remission (n = 7), and resting (n = 4) stage AAU patients is. (**B**) Average serum S100A8 levels of AAU (n = 23) compared to control (n = 29). Mean ± SE are shown. *P* values determined using One Way ANOVA, Tukey’s *post-hoc* test.

S100A8 proteins are produced intracellularly in circulating leukocytes, and then released extracellularly. To determine if S100A8 may be useful as a tool to monitor inflammatory activity in AAU, we measured the levels of S100A8 protein in serum of patients by ELISA. As shown in Table 1, a total of one-hundred samples were used in this study, which included seventy-one AAU patients and twenty-nine normal controls. Fifty-seven patients were associated with HLA-B27, and forty-one patients associated with systemic diseases including ankylosing spondylitis and Reiter’s syndrome. In contrast to our animal data, which consistently showed upregulation, the serum levels of S100A8 in AAU patients in acute, remission and resting stages were 38%, 33% and 21%, respectively, and others were below detectable levels by ELISA. This may be due to inadvertent inclusion of samples from patients who were likely to have received systemic steroid therapy before collection of blood samples. In our patient data set, an average of approximately three to five years had passed since the patient’s initial diagnosis and more than half of these patients associated with systemic diseases, both factors which increase the likelihood of previous exposure to steroids (Table 1). However, of the patients with detectable upregulation, we can clearly see significant increase with the average values of S100A8 levels found to be 237.14 ng/ml,140.87 ng/ml, and 102.53 ng/ml in the acute, remission and resting stages, respectively, while the control group average protein level was only 0.65 ng/ml (Fig. 6A). Figure 6B shows increased levels of serum S100A8 in all acute, remission, and resting stages of AAU (184.4 ng/ml compared to control, 0.65 ng/ml).

## Discussion

Uveitis and its sequelae remain a prominent cause of blindness worldwide. In many cases, the cause is unknown. Uveitis is usually an isolated illness, but it is not a single disease. Similar to arthritis, uveitis can be a part of many different disease processes. Onset of uveitis can broadly be described as a failure of the ocular immune system, and the disease results from inflammation and tissue destruction^36^. AAU is generally recognized as the most common form of uveitis. An association with HLA-B27 is seen in approximately half of AAU cases. HLA-B27 AAU can be associated with ocular inflammation alone or in association with systemic disease^37^. Migration and infiltration of cells into the eye occurs in many ocular diseases. Infiltrating granulocytes and monocytes/macrophages cause destruction by the release of chemokines, reactive oxygen species, and nitrogen, but these also regulate inflammation^38^,^39^. Several chemokines in different forms of uveitis are produced by both resident and infiltrating cells, strongly suggesting a prominent role for these molecules in its pathogenesis. Importantly, chemokines and their receptors may act as novel therapeutic targets in mediating leukocyte migration, activation and retention in inflamed ocular tissue^31^,^32^,^38^. S100A8 and S100A9 are widely-studied proteins in inflammatory disease, and in our previous study we confirmed S100A9, an essential part of the dimer with S100A8, to have a contributory function in EIU^35^. We are confident that S100A8 has a similar function and mechanism in uveitis, but this mechanism requires further confirmation.

The endotoxin-induced uveitis model was developed in rats by systemic injection of lipopolysaccharide to induce AAU^29^. It is widely used by drug companies in the development and testing of topical anti-inflammatory agents, as well as in the investigation of molecular mechanisms of ocular inflammation and for drug evaluation^31^,^32^,^35^,^36^. S100A8 reportedly has pro- and anti- inflammatory properties including chemotaxis, adhesion, and migration^19^,^20^,^21^,^22^,^23^,^24^. In the present study, the percentage of S100A8-positive granulocytes in the blood reached a maximum at 12 hours (Fig. 2C), likewise S100A8 protein levels in the circulating leukocytes reached a maximum at 12 hours after LPS injection (Fig. 1A), suggesting that the S100A8 proteins are highly expressed in granulocytes. In fact, protein levels of S100A8 were higher in granulocytes than in monocytes (Fig. 2D). Our results are consistent with the findings of Edgeworth *et al*.^3^, who reported that neutrophils contain about 40-fold more S100A8 proteins than monocytes. Since 10% is the normal granulocyte count for male Wistar rats, according to data from Sankyo Labo Service (Tokyo), it is conceivable that only 5% of the cells expressed S100A8 protein in untreated rats in the present study (Fig. 2C). Migration of leukocytes from the blood into extracellular tissue is a critical event in the pathogenesis of inflammation. S100A8 mRNA in circulating blood leukocytes reached maximum at 9 hours after LPS injection (Fig. 1A), while that in the iris-ciliary body showed a maximum level at 24–36 hours post-injection (Fig. 1B). Moreover, S100A8 in the granulocytes increased at 9 hours after LPS injection (Fig. 2D), while a few S100A8-positive cells were found in the iris-ciliary body at 18 hours and increased to their maximum at 24–36 hours (Fig. 3B). These data indicate that the migration of S100A8-positive granulocytes from the blood into the iris-ciliary body takes more than 12 hours. It has also been demonstrated that S100A8 was induced early (temporally) in monocyte/macrophage populations by LPS *in vitro,* and that S100A8 positive cells accumulated at 12 days in the iris-ciliary body in rats with EAU^40^,^41^. This supports our results, demonstrating that S100A8 expressed both in circulating blood and iris-ciliary body monocytes, but later in the granulocytes (Figs 2 and 3). Anti-rat CD14, CD68, and ED2 were used in this study to detect monocytes^42^, monocytes/macrophages^43^, and resident tissue macrophages^44^, respectively. Cells expressing S100A8 also co-express CD68, indicating S100A8-positive monocytes and macrophages in the iris-ciliary body at 36–48 hours after LPS injection (Fig 3K,L). It is likely that S100A8-positive granulocytes and monocytes/macrophages may play a role in the late phase of EIU and keratitis. ED2-positive cells did not express S100A8 in the iris-ciliary body and cornea. Our findings of resident tissue macrophage in EIU were similar to those described previously^44^. In addition, Vandal *et al*.^45^ showed that the systemic injection of anti-S100A8 and anti-S100A9 antibodies suppressed LPS-induced neutrophil migration. Our previous study also determined that intraperitoneal injection of anti-S100A9 antibody reduced EIU^35^. Numerous studies have determined that infiltration of inflammatory cells during LPS-induced anterior uveitis play a contributory role in the disease pathology^31^,^32^,^35^. In the present study, increased S100A8-positive granulocytes and monocytes have been shown in the iris-ciliary body and cornea, which have migrated and infiltrated from circulating blood in EIU rats. Interestingly, a recent study has shown that extracellular S100A8 and S100A9 are regulators of β2 integrin-dependent neutrophil slow rolling and adhesion^21^. The above findings suggest that S100A8 may be involved in the pathogenesis of EIU via promotion of migration of circulating blood leukocytes.

Glucocorticoids are widely used for treatment of uveitis in patients. Herbert *et al*. reported that DEX reduced inflammation in EIU^46^. Ishikawa *et al*. showed that glucocorticoids inhibited the migration of leukocytes into extravascular fluid^47^. According to our previous study^35^, DEX (1 mg/kg) was injected intraperitoneally 30 min before LPS in this study. Our results showed that DEX, given to EIU rats, modestly increased the levels of S100A8 mRNA and protein in blood leukocytes after LPS treatment (Fig. 4A,C), while it simultaneously inhibited S100A8-positive cell infiltration into the iris-ciliary body (Fig. 4B,E). Hsu *et al*. showed that glucocorticoids amplified the LPS-induced production of S100A8 in murine macrophages^25^. Our findings of increased S100A8 expression in leukocytes treated with DEX reinforce these results. Immunofluorescent histochemistry, performed after blood perfusion in the present study, revealed S100A8-positive cells outside of the vascular lumen. The S100A8/S100A9 protein complex binds arachidonic acid in neutrophils^48^. During neutrophil activation, S100A8/S100A9- arachidonic acid complex is transported from the cytosol to the membrane^49^. Glucocorticoids inhibit phospholipase A2 to reduce the production of arachidonic acid^50^. It is possible that DEX may inhibit transport of the S100A8/S100A9-arachidonic acid complex, resulting in suppression of neutrophil activation. This is supported by our data which suggests the possibility that DEX did not inhibit S100A8 production in the leukocytes, but rather strongly inhibited migration and infiltration of inflammatory cells from the blood into the iris-ciliary body. Notably, numerous complications of long-term steroid therapy have been reported previously, which include glaucoma and cataract. This is important to consider when deciding how and when to treat with steroids. Therefore, our results reference a valuable alternative treatment for uveitis.

The NF-kB pathway is strongly involved in EIU models, as shown extensively in the literature^31^,^32^,^35^,^51^,^52^,^53^,^54^. Leukocyte adhesion and migration is mediated by intercellular adhesion molecule-1, vascular cell adhesion molecule-1, and E-selectin, which act as counter-receptors for leukocyte β2-integrins^53^,^54^. The adhesion molecules are expressed via NF-kB pathway in vascular endothelial cells^54^. S100A8 proteins regulate adhesion of blood leukocytes through β2-integrin and NF-kB activity^19^,^20^,^21^. BAY 11-7085, an inhibitor of NF-kB activity, is widely used in animal studies^51^,^52^. As determined in our previous study, we consistently used 10 mg/kg of BAY 11-7085 in this study. Our current work shows that BAY 11-7085 suppressed S100A8 mRNA levels in leukocytes and in the iris-ciliary body, and caused suppression of inflammation in EIU rats (Fig. 5). It is possible that the NF-kB pathway is involved in S100A8 expression and the migration and infiltration of S100A8-positive cells.

Cytokine levels in blood and ocular fluids, intracellular cytokines, and cytokine gene polymorphisms in blood have all been used, with varying degrees of success, in attempts to identify patients with severe disease that is more likely to result in visual loss^36^. Vogl *et al*. demonstrated that the alarmin S100A8/S100A9 serves as a sensitive local and systemic marker for the detection of inflammatory activity^55^. Increased S100A8/A9 and S100A12 levels are found in the serum and aqueous humor of patients with autoimmune uveitis, and authors indicated that the serum levels reflect activity of joint and eye disease^27^. Pepple *et al*. also reported that S100A8 and S100A9 proteins were elevated in the aqueous and vitreous humor in both of EAU and PMU rat models^28^. So far, however, the exact extracellular functions of S100A8 protein in AAU patients have not been made clear. Our EIU study demonstrated that S100A8 has a similar function as S100A9, but the practicable clinical meaning for this remains to be determined. Our data indicates that S100A8 protein levels increase significantly in acute, remission, and even resting stages compared to the control group. In addition, our work shows the level of S100A8 expression was synchronized with AAU progression (Fig. 6). The finding of this study confirmed our hypothesis and gives us great confidence in the extension to clinical and translational research. Some problems and limitations to the study, however, remain to be addressed or considered. It is difficult for clinical researchers to obtain proper untreated samples, because AAU has a great risk of causing patient blindness without immediate treatment. Despite pre-inclusion patient interview, it is difficult to confirm that patients have not previously received systemic steroid therapy. This is due to the fact that most of our recruited patients are approximately three to five years past their initial diagnosis, and of these over half have concurrent systemic diseases such as ankylosing spondylitis (Table 1), making them even more likely to have received steroid therapy at some point. Although topical application of DEX has supposedly low systemic uptake, concentration of blood glucose levels has been shown to be increased in diabetic patients receiving DEX^56^,^57^. Therefore, if a patient has received systemic steroid therapy prior to collection of blood samples, we cannot rule out potential impact on our results. Moreover, as it is known, cultivating white blood cells can increase their secretion of many types of protein. These limitations must be taken into consideration in further studies. In multiple-factor analysis, there was no significant difference in S100A8 serum levels found in subgroup analysis for sex, age and age at onset of uveitis (data not shown). Future clinical research with a larger number and more normative samples are needed.

In summary, both intra- and extra-cellular S100A8 may play a prominent role in the pathogenesis of AAU. We report that elevated S100A8 reflects activation of granulocytes and monocytes, and inhibition of NF-kB signaling reduces circulating blood leukocyte S100A8 expression and uveitis. DEX may inhibit the migration of S100A8-positive granulocytes and monocytes from the blood into extravascular uveal tissue. S100A8 can be useful for monitoring inflammatory activity in AAU.

## Materials and Methods

### Animals

All animal experiments were conducted in compliance with the ARVO Statement for the Use of Animals in Ophthalmic and Vision Research. The study was approved by Committee of the Eye Hospital of Wenzhou Medical University for the Ethics of Animal Care and Treatment. Male Wistar rats (6–7 weeks old, 150–170 g, Charles River Laboratories, Boston, MA) were used for all animal experiments. The animals were housed under 12-hour dark and 12-hour light conditions and were given food and water ad libitum during the experiment. No repeated experiments of LPS injection in the same rats were performed.

### Antibodies

Goat anti-rat S100A8, mouse anti-rat granulocyte (HIS48)-FITC, mouse anti-rat macrophage subset (HIS36)-FITC (ED2 antigen), goat anti-rat CD68, rabbit anti-rat CD14, bovine anti-goat IgG-TR, bovine anti-goat IgG-PE, bovine anti-goat IgG-FITC, bovine anti-rabbit IgG-FITC, and normal IgG were obtained from Santa Cruz Biotechnology (Santa Cruz, CA, USA).

### EIU model and drug administration

To produce inflammation, LPS (*Escherichia coli*, serotype 055: B5, from Sigma Chemicals, St. Louis, MO, USA), 150 μg/150 μl of pyrogen-free saline, was injected intraperitoneally. Rats treated with saline served as the control. DEX (Dexamethasone, from Wako Pure Chemical, Osaka, Japan) was dissolved in 0.5% polysorbate 80. One mg/kg of the steroid was injected intraperitoneally 30 minutes before administration of LPS. Polysorbate 80, 0.5%, served as control (vehicle). BAY 11-7085, an inhibitor of I-kB phosphorylation, was obtained from Wako Pure Chemical (Osaka, Japan), and the dose of 10 mg/kg was injected intraperitoneally at the same time as LPS injection. Control animals were injected with vehicle (polyethylglycol 400 diluted 1:5 in 5% BSA/H_2_O). Rats were anesthetized with an intraperitoneal injection of pentobarbital (50 mg/kg) for blood collection via cardiac puncture.

### Real-time PCR

Blood was collected in an EDTA-coated syringe from the right atrium in rats. Leukocytes were isolated using SV RNA red blood cell lysis solution (Promega, Madison, WI), and total RNA was extracted using the SV total RNA isolation system (Promega). The eye globe was enucleated and immediately submerged in RNA stabilization reagent (Qiagen, Hilden, Germany). Next, the iris-ciliary body tissue was isolated from the stabilized eye, and then homogenized with a rotor- stator homogenizer in buffer RLT (Qiagen). Total RNA was extracted using an RNeasy Protect Mini Kit (Qiagen) and treated with RNase- free DNase Set (Qiagen) to remove any residual genomic DNA. cDNA from each sample was obtained by reverse transcription with random hexamers using MultiScribe reverse transcriptase (Thermo Fisher Scientific, Waltham, MA). Real-time PCR were performed using TaqMan probes and TaqMan Universal Master Mix, and detected by ABI PRISM 7700 system (Thermo Fisher Scientific, Waltham, MA). To compare expression pattern, mRNA template concentrations for GAPDH and the target genes were calculated using the standard curve method. The expression level of S100A8 mRNA was normalized by GAPDH mRNA level in each sample, and the changes were expressed as an n-fold increase relative to the value of rats treated with saline.

### Immunohistochemistry

The blood was washed with phosphate-buffered saline (PBS) and the animals were perfused with 4% cold paraformaldehyde (PFA) in PBS at 0, 12, 18, 24, 36, 48, or 72 hours after LPS injection. Then, the eyes were enucleated, cut in half, and post fixed with 4% PFA in PBS for 20 minutes at 4 °C. Specimens were OCT-embedded, frozen, and cryosectioned at 10 micrometer thickness. The sections underwent double-labeling immunofluorescence staining. After blocking with 10% normal bovine serum in PBS, slides were incubated with goat anti-rat S100A8 polyclonal antibody (Santa Cruz Biotechnology, Santa Cruz, CA, USA) for 1 hour and immunolabeled with Texas Red conjugated bovine anti-goat IgG secondary antibody for 30 minutes. Next, the sections were incubated with FITC-conjugated mouse anti-rat granulocyte monoclonal antibody or mouse anti-rat macrophage subset-FITC for 1 hour, or incubated with goat anti-rat CD68 polyclonal antibody for 1 hour and immunolabeled with FITC conjugated bovine anti-goat IgG secondary antibody for 30 minutes. Negative controls were incubated with normal bovine IgG or primary antibody (data not shown).

### Flow cytometric analysis

Venous blood of rats was collected in EDTA-coated syringes at 0, 6, 9, 12, 18, 24, 36, or 48 hours after LPS injection. White blood cells were immediately isolated and then a flow cytometric assay of S100A8 expression was performed as described previously^35^. For membrane staining, 1 × 10^6^ cells were incubated with mouse anti-rat granulocyte-FITC or with rabbit anti-rat CD14 primary antibody and bovine anti-rabbit IgG-FITC. For cytoplasmic staining, after being fixed in 4% PFA and permeabilized with 0.1% Triton X-100, the cells were blocked with 20% normal bovine serum in PBS. The cells were incubated with goat anti-rat S100A8 polyclonal antibody and then stained with bovine anti-goat IgG-PE. The cells were analyzed in a Becton Dickinson FACS Calibur FCM using Cell Quest software. Analyses of results were performed with at least 10,000 cells per sample. The results were expressed as the log of fluorescent intensity.

### Measurement of total protein and cell number

Aqueous humor was aspirated 24 hours after LPS injection using a 30-G needle with visualization under a microscope. Aspirated samples were centrifuged at 1,500 rpm for 5 min at 4 °C to obtain the supernatant. The protein concentration in the supernatant was measured by a protein-dye binding assay as previously described^31^,^32^ and was expressed relative to a bovine serum albumin standard. The aspirated aqueous humor was mixed with a 1:1 volume of trypan blue, and cell number counted using a hematocytometer.

### Patients

All patients involved in this study were treated according to the tenets of the Declaration of Helsinki and was approved by the Clinical Research Ethics Committee at the Eye Hospital of Wenzhou Medical University, China. Written informed consent was obtained from all subjects.

Seventy-one patients with AAU were recruited to the study from 2014 to 2015. AAU, diagnosed as iridocyclitis or iritis with less than 3 months duration, is an inflammatory disorder of the anterior part of the uvea, which may be associated with a number of disease entities^58^. In order to assess the progression of AAU, we classified AAU patients into three stages, according to the inflammation score (Table 2): acute (score 3), remission (score 2), or resting stage (score 0–1). During the patient blood sample collection period, we gave topical treatments to the patients according to their clinical specificity. For cases in the acute and remission phases, dexamethasone injected under the ball subconjunctival or TobraDex eye ointment was administered, and eye drops of nonsteroidal anti-inflammatory drugs (NSAIDs) were administered for patients in the resting stage. The exclusion criteria included infectious uveitis or patients with a pre-existing ocular disease such as keratoconjunctivitis, glaucoma, retinopathy, maculopathy, previous ocular surgery, trauma, etc. Twenty-nine people without ocular inflammation and systemic autoimmune diseases served as controls.

### ELISA

Venous blood samples of patients were obtained using BD Vacutainer plasma tubes (Heparin) and centrifuged at 1200 g for 10 minutes within an hour after acquisition. The serum aliquots were stored at −80 °C until analysis. Protein levels of serum S100A8 were determined by specific double antibody sandwich enzyme-linked immunosorbent assay (ELISA) systems as previously described^31^,^32^. The Human S100A8 DuoSet ELISA kit was purchased from R&D systems (Minneapolis, MN, USA) and measurement concentrations were prepared according to the manufacturer’s instructions.

### Statistics

All animal data were analyzed and presented throughout as the mean ± standard deviation (SD). The clinical data was analyzed and plotted using GraphPad Prism software and presented as the mean ± standard error of the mean (SEM). Statistical analyses were performed by *t*-test or Tukey’s *post-hoc* test after One-way ANOVA for multiple comparisons of mean values. P < 0.05 was considered statistically significant.

## Additional Information

**How to cite this article**: Wang, Y. *et al*. S100A8 promotes migration and infiltration of inflammatory cells in acute anterior uveitis. *Sci. Rep.* **6**, 36140; doi: 10.1038/srep36140 (2016).

**Publisher’s note:** Springer Nature remains neutral with regard to jurisdictional claims in published maps and institutional affiliations.

## Figures and Tables

**Table 1:** General characteristics of the study population.

	acute	remission	resting	control
Patients, *n*	31	21	19	29
Patient age, mean ± SD	43.42 ± 15.26	43.09 ± 13.71	42.56 ± 13.70	36.36 ± 10.45
Age at uveitis diagnosis, mean ± SD	38.59 ± 16.01	37.60 ± 14.32	39.31 ± 12.67	
Duration of AAU	4.83	5.49	3.25	
Grading score	3	2	0–1	0
Male sex, *n* (%)	22 (71.0)	13 (61.9)	14 (73.7)	19 (65.5)
HLA-B27, *n* (%)	24 (77.4)	19 (90.5)	14 (73.7)	
Any ocular complications, *n* (%)	16 (51.6)	12 (57.1)	13 (68.4)	
Active uveitis, *n* (%)	31 (100)	11 (52.4)	0	

**Table 2:** Grading scheme for inflammatory activity of AAU^59^.

Scores	0	1	2	3
Symptoms				
Ciliary congestion	none	mild	moderate	severe
KP	0~+	+~++	++~+++	+++~++++
AC cell	0	0~++	++~+++	+++~++++
AC flare	0~+	0~++	++~+++	+++~++++
Others	−	−	−	Empyema and Exudation

KP: keratic precipitates; AC: anterior chamber.
